# Managing Rectal Adenocarcinoma in a Radiation-Exposed Patient: A Case Report Utilizing the PROSPECT Trial Protocol

**DOI:** 10.7759/cureus.105786

**Published:** 2026-03-24

**Authors:** Talal Al-Assil, John Han, Evan Kim, John Seneriches, Nazmul Hasan

**Affiliations:** 1 Oncology, UCI Medical Center, Orange, USA

**Keywords:** cervical cancer, colorectal surgery, neoadjuvant chemotherapy (nact), oncology radiation, rectal adenocarcinoma

## Abstract

This report describes a 76-year-old woman with newly diagnosed rectal adenocarcinoma and a complex oncologic history, including stage IV cervical cancer previously treated with pelvic chemoradiation, which resulted in significant long-term complications. Given her prior extensive radiation exposure, standard neoadjuvant chemoradiation posed substantial toxicity risks. A chemotherapy-first approach, informed by the PROSPECT trial protocol, was therefore implemented. The patient received neoadjuvant FOLFOX chemotherapy followed by surgical resection, achieving tumor regression and favorable pathological outcomes without additional pelvic radiation. This case illustrates the potential role of a PROSPECT-informed, radiation-sparing strategy in carefully selected patients with locally advanced rectal cancer and prior pelvic irradiation, highlighting the importance of individualized, multidisciplinary treatment planning in complex oncologic scenarios.

## Introduction

Rectal adenocarcinoma accounts for a substantial portion of colorectal cancers, the fourth most common cancer worldwide, often demanding comprehensive and multimodal therapies [1,2]. Due to the anatomic location of the rectum within the confined space of the pelvis and its proximity to radiosensitive organs, treating rectal tumors poses unique technical and toxicity-related challenges [3]. Standard management of locally advanced rectal cancer (LARC) typically involves neoadjuvant chemoradiation therapy or total neoadjuvant therapy (TNT) followed by total mesorectal excision (TME), with radiation playing a key role in optimizing local control, especially in cases of significant lymph node involvement or local spread [4-6].

While pelvic radiation is frequently employed in patients with LARC, its cumulative doses are associated with significant acute and long-term gastrointestinal and genitourinary toxicities [7]. In fact, moderate-to-severe urinary toxicity has been observed in 80% of patients exposed to pelvic radiation [8]. These concerns are particularly relevant in patients with prior pelvic radiation. Although re-irradiation may be a viable option in select patients, it carries heightened risks due to reduced normal-tissue tolerance, uncertain cumulative dose thresholds, and limited prospective safety data [9].

The recent PROSPECT trial evaluated whether neoadjuvant FOLFOX chemotherapy with selective radiation could provide disease control comparable to standard chemoradiation in patients with LARC. In this phase III randomized trial of 1,194 patients with stage T2N+, T3N-, or T3N+ LARC, neoadjuvant FOLFOX with response-adapted selective chemoradiation for inadequate responders was found to be non-inferior to chemoradiotherapy in terms of disease-free survival, offering a potential strategy to omit pelvic radiation in appropriately selected patients [10].

This evolving paradigm is especially relevant in individuals with multiple cancers, prior extensive pelvic radiation exposure, and complex medical backgrounds, in whom standard chemoradiation may pose prohibitive toxicity risks. We present this case of a 76-year-old woman with a history of chemoradiation for cervical cancer who was subsequently diagnosed with T3N1M0 rectal adenocarcinoma and treated according to a radiation-sparing total neoadjuvant approach consistent with the PROSPECT protocol.

## Case presentation

The patient was a 76-year-old female with newly diagnosed rectal adenocarcinoma. Her medical history included stage IV cervical cancer with distant lymph node metastasis, which had been treated in 2006 with chemoradiation and brachytherapy, resulting in remission by mid-2007. Her radiation treatment in 2006 included external beam radiation therapy (EBRT) at 45 Gy in 25 fractions, a boost of 10 Gy, and brachytherapy at 30 Gy, for a total pelvic dose of 85 Gy. In 2008, a subsequent colonoscopy revealed radiation proctitis, and she experienced chronic diarrhea and fecal incontinence.

A follow-up colonoscopy scheduled for 2018 had been delayed due to other medical conditions, including bilateral hydronephrosis and small bowel obstruction (SBO). Her hydronephrosis was chronic and bilateral, secondary to radiation-induced ureteral fibrosis, and was managed with nephrostomy tubes until October 2022, when ileo-urinary conduit surgery was determined to be required. She had also experienced intermittent episodes of SBO prior to the conduit, likely radiation-induced.

Diagnosis of rectal adenocarcinoma was made in November 2023 via colonoscopic biopsy, which revealed an ulcerated lesion with exudate covering three-fourths of the distal rectum. Histologic evaluation confirmed moderately differentiated invasive adenocarcinoma (Table 1). Immunohistochemical staining (Block D) showed CK20 and CDX2 positivity with CK7, CK5/6, ER, and p63 negativity, supporting a colorectal origin. Mismatch repair proteins (MLH1, PMS2, MSH2, and MSH6) demonstrated retained nuclear expression, consistent with a mismatch repair-proficient tumor (Table 2).

**Table 1:** Final pathology report from colonoscopy biopsy

Specimen	Site	Diagnosis
A	Colon (random biopsy)	Benign colonic mucosa; negative for microscopic colitis, chronic changes, granulomas, dysplasia, or malignancy
B	Hepatic flexure polyp	Tubular adenoma
C	Transverse colon polyp	Tubular adenoma
D	Anal rectal ulcerated mass	Moderately differentiated adenocarcinoma; mismatch repair proficient

**Table 2:** IHC results of rectal mass (specimen D) IHC, immunohistochemistry

Marker	Method	Result
CK5/6	IHC	Negative
CK7	IHC	Negative
CK20	IHC	Tumor positive
CDX2	IHC	Tumor positive
ER	IHC	Negative
p63	IHC	Negative
PMS2	IHC	Retained nuclear expression
MLH1	IHC	Retained nuclear expression
MSH2	IHC	Retained nuclear expression
MSH6	IHC	Retained expression

**Figure 1:**
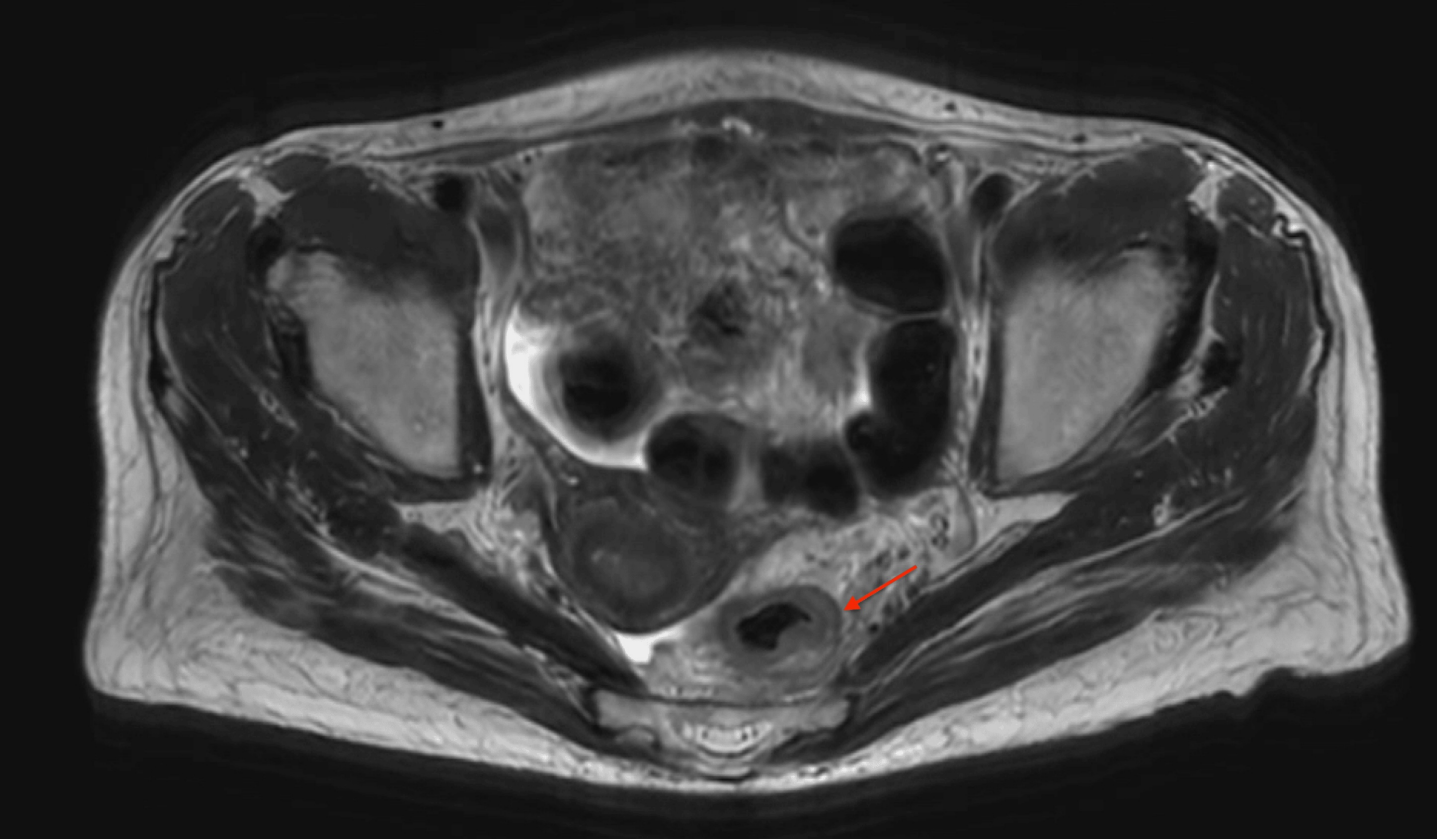
Axial T2-weighted pelvic MRI demonstrating asymmetric rectal wall thickening (arrow) at the site of the primary tumor, consistent with T3 disease These findings necessitated consideration of multimodal therapy and posed a clinical dilemma regarding the addition of pelvic radiation in a patient with prior radiation exposure.

The staging pelvic MRI report showed rectal adenocarcinoma, demonstrated by tumor extension beyond the muscularis propria into the mesorectal fat, involving three-fourths of the distal rectal circumference and regional mesorectal lymph nodes. There was no evidence of distant metastasis or local invasion of adjacent organs, indicating T3N1M0 disease (Figure 1).

Given her previous extensive radiation exposure and ongoing issues with radiation-induced damage, she was treated with a chemotherapy regimen without radiation based on the PROSPECT trial. In December 2023, the patient initiated FOLFOX chemotherapy. The final dose was administered on January 31, 2024, after she completed four cycles. Severe diarrhea complicated the chemotherapy, necessitating two hospitalizations in February 2024 and a prolonged recovery period. As a consequence, chemotherapy was suspended to facilitate healing. The patient subsequently reported substantial improvement and successfully completed cycles five and six by April 2024.

Post-treatment MRI demonstrated an approximately 20% decrease in tumor size compared with baseline, with reduced rectal wall thickening, consistent with a partial radiographic response. No evidence of distant metastatic disease was identified. In June 2024, the patient underwent a robotic abdominoperineal resection (APR). Following surgical intervention, pathological staging revealed ypT2N0 disease, with 0/14 lymph nodes positive, no evidence of angiolymphatic or perineural invasion, and negative margins, all consistent with treatment response. The tumor was also determined to be microsatellite stable.

**Figure 2:**
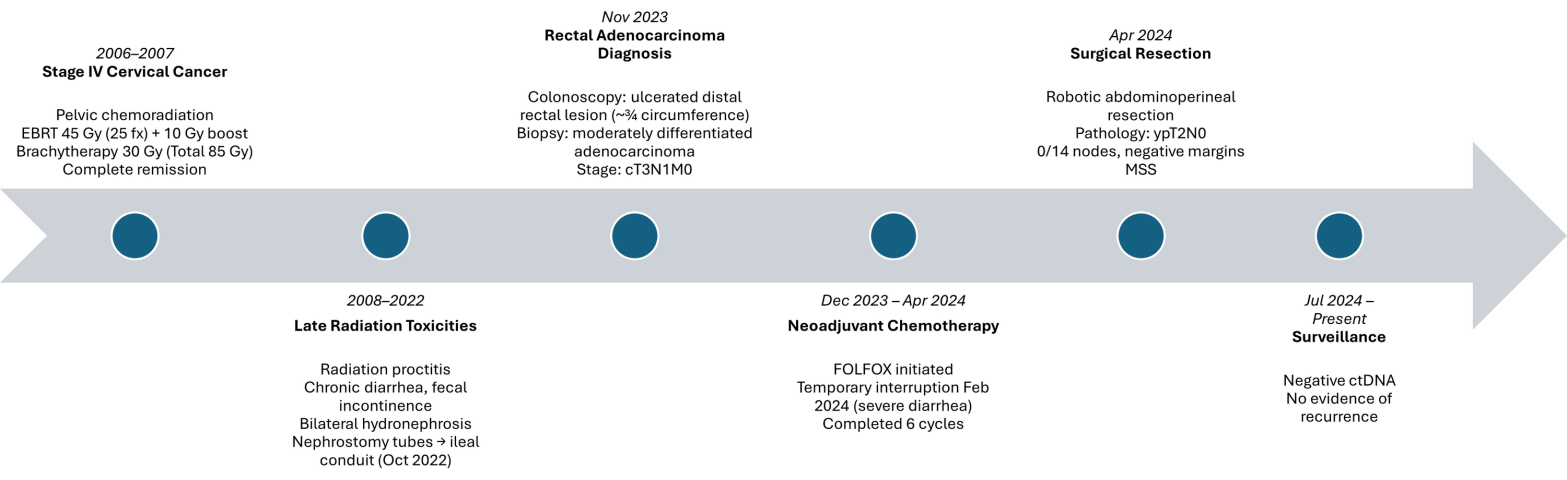
Clinical timeline of prior cervical cancer treatment and subsequent rectal adenocarcinoma management EBRT, external beam radiation therapy; MSS, microsatellite stable This image was created using Microsoft PowerPoint.

The patient made a full recovery postoperatively, and there were no indications of local or distant recurrence. As of the latest follow-up, her ctDNA remained negative, further suggesting the absence of residual disease. A clinical timeline summarizing the patient’s course is shown in Figure 2.

## Discussion

Rectal adenocarcinoma is commonly managed using a multimodal treatment strategy, particularly in cases of locally advanced disease. Current guidelines recommend TNT, which combines systemic chemotherapy (such as FOLFOX or CAPOX) with chemoradiation, followed by surgical resection [11,12]. This approach has been shown to improve pathological response rates, local control, and sphincter preservation. However, treatment strategies may vary depending on tumor stage, tumor location, and patient-specific factors.

Radiation therapy has been a cornerstone of rectal adenocarcinoma management for several decades. Multiple landmark randomized trials have evaluated the role of radiation in the neoadjuvant setting (Table 3), consistently demonstrating improved local control and reductions in local recurrence rates. Across these studies, however, the overall survival advantage of preoperative radiation has been inconsistent, with select studies, such as the TME trial, demonstrating subset-specific overall survival benefits [13]. This observation has prompted increasing interest in optimizing systemic therapy to address distant metastases, which remain a major determinant of long-term prognosis [10-14].

**Table 3:** Summary of key studies on radiation therapy in neoadjuvant and adjuvant treatments for rectal cancer LARC, locally advanced rectal cancer; TME, total mesorectal excision

Study	Author	Year	Objective	Methods	Results	Conclusion
PROSPECT trial	Schrag et al. [10]	2023	Compare neoadjuvant FOLFOX to chemoradiotherapy for LARC	Multicenter, randomized trial; FOLFOX vs. chemoradiotherapy	FOLFOX was noninferior to chemoradiotherapy in disease-free survival; similar overall survival and local recurrence	FOLFOX-first with selective radiation is a viable alternative to routine chemoradiotherapy for LARC
SAJC study	Thakur et al. [11]	2020	Compare long-course chemoradiotherapy with short-course radiotherapy followed by chemotherapy	Prospective observational study; short-course radiotherapy vs. long-course chemoradiotherapy	Similar pathological complete response rates; lower acute toxicity in short-course radiotherapy	Short-course radiotherapy with delayed surgery may reduce toxicity and improve compliance
MRC CR07/NCIC-CTG C016	Sebag-Montefiore et al. [12]	2009	Compare preoperative radiotherapy to selective postoperative chemoradiotherapy	Randomized trial; preoperative radiotherapy vs. postoperative chemoradiotherapy	Preoperative radiotherapy reduced local recurrence and improved disease-free survival	Preoperative radiotherapy is effective for operable rectal cancer
TME trial	Van Gijn et al. [13]	2011	Evaluate preoperative radiotherapy combined with TME	Randomized trial; preoperative radiotherapy vs. surgery alone	Preoperative radiation led to an overall survival benefit for clinical stage III with negative margins at the time of surgery	Overall, preoperative radiotherapy improves local control, but overall survival is inconsistent
NSABP R-01	Fisher et al. [14]	1988	Evaluate adjuvant chemotherapy or radiotherapy for rectal cancer	Randomized trial; surgery alone vs. postoperative radiotherapy vs. adjuvant chemotherapy	Chemotherapy improved disease-free survival and overall survival in males; radiotherapy reduced local recurrence but not disease-free survival or overall survival	Adjuvant chemotherapy benefits males; radiotherapy reduces local recurrence
CAO/ARO/AIO-94	Sauer et al. [15]	2012	Compare preoperative vs. postoperative chemoradiotherapy for LARC	Randomized trial; preoperative vs. postoperative chemoradiotherapy	Improved local control with preoperative chemoradiotherapy; no overall survival benefit	Preoperative chemoradiotherapy improves local control but not overall survival

**Figure 3:**
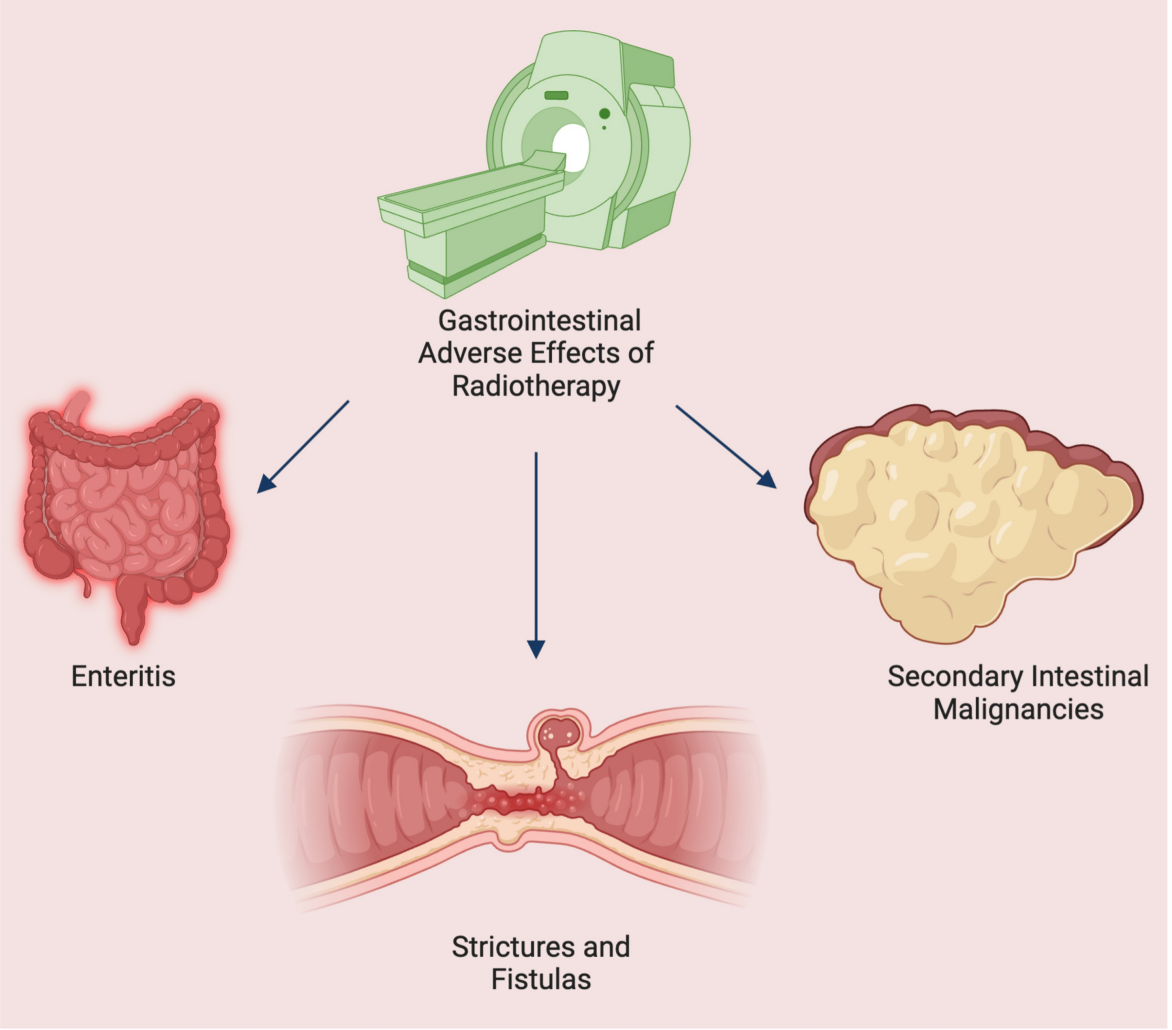
Overview of major gastrointestinal toxicities associated with pelvic radiation, including enteritis, strictures, fistulas, and secondary malignancies These cumulative risks are central to treatment decision-making in patients with prior pelvic irradiation. This image was created using BioRender.

Although radiation therapy can improve local tumor control and surgical outcomes, its routine use in all patients with LARC has been questioned. Increasing attention has been directed toward treatment strategies that emphasize effective systemic therapy while limiting treatment-related toxicity. Radiation therapy is associated with a range of potential complications, particularly when delivered to the abdomen and pelvis. These may include enteritis, strictures, fistula formation, and, less commonly, secondary malignancies within the irradiated field (Figure 3) [16]. Thus, while radiation improves local control, its overall survival benefit is limited and may introduce additional morbidities.

These considerations become especially important in patients with complex oncologic histories. In our patient, a prior diagnosis of stage IV cervical cancer treated with pelvic chemoradiation significantly complicated the management of her subsequent rectal adenocarcinoma. She experienced long-term radiation-related toxicities, including radiation proctitis, chronic diarrhea, and genitourinary complications. Furthermore, it is possible that her prior pelvic radiation contributed to the later development of rectal cancer [17]. Radiation exposure is cumulative over a lifetime, and exceeding normal tissue tolerance substantially increases the risk of severe complications [18]. Thus, while re-irradiation of the abdomen and pelvis can be technically feasible, it requires careful evaluation of cumulative radiation dose, patient comorbidities, and the tolerance of surrounding organs.

Advances in radiation techniques, including stereotactic body radiation therapy, intensity-modulated radiation therapy, and image-guided radiation therapy, have improved the ability to deliver highly conformal radiation while sparing adjacent tissues [19]. Moreover, radiation-induced fibrosis and reduced tissue vascularity may impair wound healing and increase the risk of postoperative complications such as infection or wound dehiscence [20]. Therefore, the decision to pursue re-irradiation must be made on an individualized basis and cautiously. In addition to the risk of toxicity, prior radiation can significantly affect surgical outcomes. Given our patient’s extensive prior radiation exposure within the surgical field, additional radiation posed substantial risks and could have further compromised her surgical candidacy.

**Figure 4:**
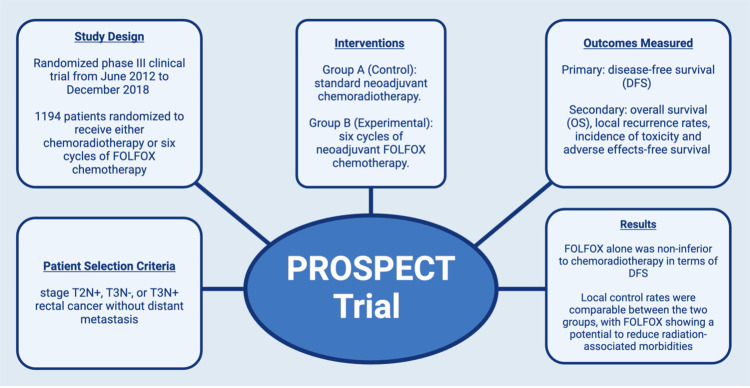
Summary of the PROSPECT trial design and outcomes demonstrating non-inferior disease-free survival with neoadjuvant FOLFOX compared to chemoradiotherapy, supporting chemotherapy-first strategies in select patients in whom radiation avoidance is clinically desirable DFS, disease-free survival; OS, overall survival This image was created using BioRender.

In this context, the PROSPECT trial protocol provided a rational treatment strategy by allowing initial neoadjuvant chemotherapy with selective use of radiation based on treatment response (Figure 4). This approach permitted avoidance of additional pelvic radiation while maintaining oncologic treatment intensity. Importantly, the PROSPECT trial still recommends radiation therapy for patients whose tumors demonstrate inadequate response to initial chemotherapy, thereby preserving the role of chemoradiation when necessary to optimize local control [21]. Such an adaptive treatment strategy acknowledges the variability in patient responses while balancing oncologic efficacy with treatment-related toxicity. PROSPECT does not establish routine omission of radiation therapy. Rather, it provides an evidence-based framework for tailoring chemoradiation based on response and patient-specific constraints. Long-term follow-up will also be important to ensure durable local control in patients managed without routine radiation.

It is also important to recognize that the PROSPECT trial excluded patients who were expected to undergo APR. This exclusion was intended to maintain a more homogeneous study population and reduce variability in surgical outcomes. Consequently, the application of the PROSPECT protocol in patients requiring APR, as in this case, falls outside the original trial design. Additionally, the trial reported a higher overall toxicity rate in the FOLFOX-first arm, underscoring the importance of individualized selection and multidisciplinary discussion. The decision to proceed with this strategy was therefore guided by the patient’s extensive prior radiation exposure and the clinical risk-benefit analysis favoring the avoidance of additional pelvic irradiation [10]. Further investigation and longer-term follow-up are needed to better understand the safety and outcomes of applying PROSPECT-based approaches in similar clinical scenarios.

Despite increasing interest in chemotherapy-first strategies, radiation therapy remains an important component of the standard treatment paradigm for LARC. Current NCCN guidelines continue to recommend radiation as part of TNT for many patients, based on decades of evidence demonstrating improved local control and reductions in local recurrence. From a radiation oncology perspective, preoperative radiation provides reliable pelvic disease control and may facilitate margin-negative resection in tumors threatening the mesorectal fascia. The PROSPECT trial, therefore, should not be interpreted as eliminating the role of radiation therapy, but rather as identifying a subset of patients in whom radiation may be safely deferred if an adequate response to systemic chemotherapy is achieved. Importantly, the trial included carefully selected patients with intermediate-risk tumors and excluded those expected to require APR, which may limit generalizability to broader rectal cancer populations.

While this case demonstrates the feasibility of a PROSPECT-informed, risk-adapted approach in a previously irradiated patient, conclusions drawn from a single case remain inherently limited in generalizability. This report does not imply superiority of chemotherapy-first strategies over established multimodal standards, nor does it advocate routine omission of radiation therapy in LARC. Rather, it illustrates how trial-informed principles may be thoughtfully adapted for highly selected patients with unique clinical constraints within a multidisciplinary decision-making framework.

## Conclusions

The application of a PROSPECT-informed treatment strategy in this patient with rectal adenocarcinoma and extensive prior pelvic radiation illustrates both the feasibility and clinical considerations of a chemotherapy-first approach in carefully selected cases. The patient demonstrated considerable progress following the PROSPECT chemotherapy protocol without the need for radiation, indicating that it could serve as a feasible alternative to conventional chemoradiation in certain patients. This case highlights the importance of individualized, multidisciplinary decision-making in patients with complex oncologic histories and underscores the continued need for prospective studies to refine treatment strategies for rectal cancer.
